# Efficacy of mesenchymal stem cell transplantation therapy for type 1 and type 2 diabetes mellitus: a meta-analysis

**DOI:** 10.1186/s13287-021-02342-5

**Published:** 2021-05-06

**Authors:** Yanju Li, Feiqing Wang, Huiling Liang, Dongxin Tang, Mei Huang, Jianing Zhao, Xu Yang, Yanqing Liu, Liping Shu, Jishi Wang, Zhixu He, Yang Liu

**Affiliations:** 1 Department of Hematology, Affiliated Hospital of Guizhou Medical University, Guiyang, 550004 Guizhou Province China; 2 National & Guizhou Joint Engineering Laboratory for Cell Engineering and Biomedicine Technique, Guizhou Province Key Laboratory of Regenerative Medicine, Key Laboratory of Adult Stem Cell Translational Research, Chinese Academy of Medical Sciences, Guizhou Medical University, Guiyang, 550004 Guizhou Province China; 3 Academy of Medical Engineering and Translational Medicine, Tianjin University, Tianjin, 300072 China; 4 Department of Scientific Research, The First Affiliated Hospital of Guizhou University of Traditional Chinese Medicine, Guiyang, 550001 Guizhou Province China

**Keywords:** Mesenchymal stem cells, Diabetes mellitus, Type 1 diabetes mellitus, Type 2 diabetes mellitus, Transplantation therapy

## Abstract

**Background:**

This meta-analysis was first conducted to evaluate the efficacy and safety of transplantation of mesenchymal stem cells in the treatment of type 1 and type 2 diabetes mellitus (T1DM and T2DM).

**Methods:**

We systematically searched PubMed, ScienceDirect, Google Scholar, CNKI, EMBASE, Web of Science, MEDLINE, and the Cochrane Library for studies published from the establishment of the databases to November 2020. Two researchers independently screened the identified studies, based on inclusion and exclusion criteria. The combined standard mean difference (SMD) and 95% confidence interval (CI) of data from the included studies were calculated using fixed- or random-effects models.

**Results:**

We included 10 studies in our meta-analysis (4 studies on T1DM and 6 on T2DM, with 239 participants) to examine the efficacy of mesenchymal stem cells (MSCs) therapy in the treatment of diabetes mellitus. According to the pooled estimates, the glycated hemoglobin (HbA1c) level of the MSC-treated group was significantly lower than it was at baseline (mean difference (MD) = −1.51, 95% CI −2.42 to −0.60, *P* = 0.001). The fasting C-peptide level of the MSC-treated group with T1DM was higher than that of the control group (SMD = 0.89, 95% CI 0.36 to 1.42, *P* = 0.001), and their insulin requirement was significantly lower than it was at baseline (SMD = −1.14, 95% CI −1.52 to −0.77, *P* < 0.00001).

**Conclusion:**

Transplantation of mesenchymal stem cells has beneficial effects on diabetes mellitus, especially T1DM, and no obvious adverse reactions.

## Background

The incidence of diabetes mellitus (DM) is increasing yearly. According to the International Diabetes Federation, 4.51 million adults worldwide were estimated to have DM in 2017, and this figure is expected to reach 6.93 million by 2045 [1]. Type 1 diabetes mellitus (T1DM) is an autoimmune disease, and immune attacks lead to the destruction of islet cells, causing islet inflammation associated with absolute insulin deficiency. Eventually, various related complications occur, causing serious harm to the patient and negative effects on the patient’s quality of life and longevity [2]. Type 2 diabetes mellitus (T2DM) is the most common type of diabetes, accounting for diagnoses in approximately 90% of adults [3]. The main pathogenesis is the body’s insensitivity to insulin and impaired functioning of islet β cells [4]. At present, neither oral hypoglycemic drugs nor insulin is a cure for diabetes; these treatments increase the risk of complications, such as hypoglycemia, gastrointestinal intolerance, heart failure, and atypical fractures [5].

Islet transplantation can theoretically cure diabetes, but it is limited by a lack of donor sources and susceptible to immune rejection complications and difficulties related to the separation of the pancreatic islets [6]. In recent years, stem cell-based transplantation has shown advantages in the treatment of diabetes. Unlike embryonic stem cells, the use of mesenchymal stem cells (MSCs) in the treatment of diabetes does not involve tumorigenic risks or ethical issues [7–9]. MSC transplantation is an attractive option due to its wide range of sources, easy access [10], self-renewal ability, multi-differentiation potential, low immunogenicity, secretion of various cytokines, and other biological characteristics, and it is not ethically controversial [11–13]. In an animal study conducted in Egypt, the blood glucose level of rats with T1DM that were injected with differentiated MSCs, returned to normal after 2 months [14]. A study by Vanikar et al. of 11 patients with T1DM reported a significant decrease in insulin requirements and HbA1c levels, and an increase in C-peptide (CP) levels after co-transplantation of insulin-secreting adipose tissue-derived MSCs and hematopoietic stem cells [15]. The injection of adipose-derived MSCs has been shown to reduce insulin resistance in rats with T2DM by inhibiting the increase in Mitsugumin 53 in the skeletal muscle [16, 17]. Bhansali et al. found that after 12 months of follow-up, participants’ level of HbA1c and insulin requirements decreased significantly, their fasting C-peptide level (F-CP) showed no significant change, and their fasting blood glucose increased [18]. Liu et al. study reported an increase in participants’ F-CP level at the end of the follow-up period, but there was no significant change in the oral glucose tolerance test CP level 2 h after a meal, compared with its baseline level [19].

Although the results of the above studies indicate that MSCs are effective in the treatment of both T1DM and T2DM, no studies comparing the efficacy of MSCs in the treatment of T1DM and T2DM have been published. Furthermore, most of the published studies had small sample sizes and did not provide sufficient validation. Therefore, our meta-analysis of the differential curative effects of MSCs on T1DM and T2DM, and their safety, was conducted to provide a theoretical basis for the clinical diagnosis and treatment of DM.

## Methods

### Data sources and search strategies

Two of the study’s authors (WFQ and LHL) searched the PubMed, ScienceDirect, Google Scholar, CNKI, EMBASE, Web of Science, and MEDLINE databases, as well as the Cochrane Library and other databases for eligible studies, up to November 2020. They consulted with the senior author when there were discrepancies in the selection of studies. The search terms in Chinese and English included “mesenchymal stem/stromal cell” or “stem/stromal cell” and “diabetic” or “type 1 diabetes” or “type 2 diabetes,” and “clinical trials.” Manual searches of the reference lists of relevant studies and narrative reviews were also performed. The search was limited to English and Chinese papers and human subjects, and published studies; unpublished studies were not included in the meta-analysis.

### Selection criteria

The inclusion criteria were (1) Chinese- and English-language research articles; (2) clinical trials on the use of MSCs for treating DM; (3) all patients with DM were treated with MSCs regardless of age, gender, disease severity, or location; and (4) all studies evaluated the treatment of DM using MSCs. There were no restrictions on the time, duration, or dosages of MSCs in the treatments. The control group received a blank treatment, placebo, or other treatment. The dose and course of all other treatments were the same as those for the MSC-treated and control groups. The exclusion criteria were (1) research studies in languages other than Chinese and English, (3) incomplete research reports or data (e.g., missing sections, such as a conference abstract), and (4) redundant publications (the most recent and complete studies, including clinical trials, were selected for inclusion in the meta-analysis).

### Data extraction

The two authors who conducted the systematic search used a standard data extraction table to collect information independently in accordance with the standards of the Cochrane Systematic Review Protocol. The data that were extracted included the first author, study characteristics (i.e., the study’s objective, year of publication, and country), participants’ characteristics (e.g., mean age, sex, sample size, and mean history of DM), and experimental design, measured outcomes (e.g., HbA1c; fasting C-peptide, F-CP; fasting blood glucose, FBG; postprandial blood glucose, PBG; and insulin requirements). Standard deviations were calculated from standard errors or confidence intervals, as needed. Changes in baseline standard deviations were calculated using the correlation coefficient method, which is described in the Cochrane Handbook for Systematic Reviews of Interventions. A third author was consulted to help resolve disagreements between the two previously mentioned authors, concerning the inclusion of studies.

### Statistical analysis

The weighted mean difference (WMD) was used to compare continuous variables when the measurement method and unit of measurement were the same for different studies; otherwise, the standardized mean difference (SMD) was used as the effect. Results reported as medians and quartiles were converted to the mean and standard deviation (SD) [20, 21]. A two-tailed value of *P* < 0.05 was considered statistically significant. We evaluated the heterogeneity of the included studies by calculating the *I*^*2*^ statistic; the *I*^*2*^ values were 25%, 50%, and 75%, indicating low, medium, and high heterogeneity, respectively. When the effects were observed to be heterogeneous (*I*^*2*^ *>*50% and *P* < 0.10), we used a random-effects model for the analysis [22]; otherwise, a fixed-effects model was used to evaluate the data. Our meta-analysis was performed using Revman 5.3 software.

## Results

### Search results

**Fig. 1:**
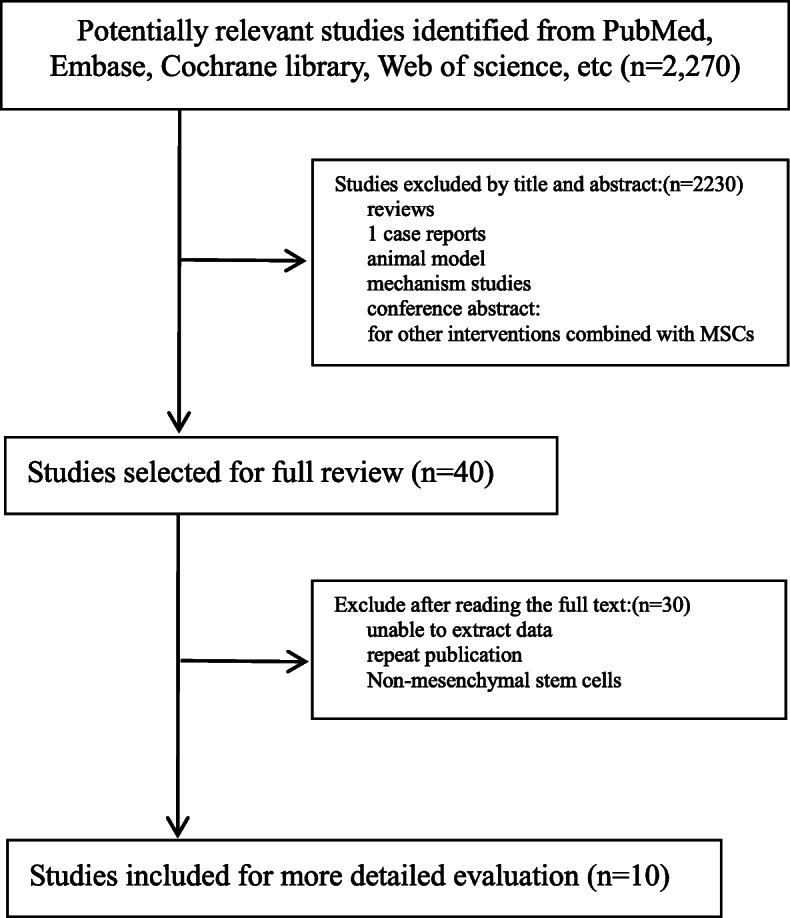
Flow diagram of the search process and study selection

Our search terms yielded 2270 potential research articles for inclusion in the study. After reading the titles and abstracts, 2230 studies were found to be irrelevant in terms of their purpose, objective, intervention, and/or measures and were excluded. After reading the remaining 40 papers, 30 were excluded. Finally, 10 clinical studies [18, 19, 23–30], consisting of 239 patients with DM, were included in the meta-analysis. There were 92 cases of T1DM and 147 cases of T2DM, six studies included a control group, 143 patients were treated with MSCs, and 96 patients served as controls. Details of the study selection process are shown in Fig. 1.

### Characteristics of the included studies

The clinical data from the included studies in Table 1 were published from 2011 through 2017, with sample sizes ranging from 6 to 61. Of these ten studies, eight were conducted in China, one in India, and one in Sweden. The intervention regimen consisted of MSCs with a dose range of (0.88 ± 0.05) × 10^6^ to (1.2 ± 0.3) × 10^9^, and the follow-up period ranged from 6 to 36 months.

**Table 1:** Baseline characteristics of the study populations in the 10 studies included in the meta-analysis

Author year	Regimen	Age (years)	Sample size (females)	BMI (kg/m^2^)	HbA1c (%)	FBG (mmol/l)	Type of diabetes	Duration of DM	Dose of injected cells	Mode of injection	Follow-up (months)
Carlsso n 2015 [23]	hBM-MSCs Control	24±6 27±6	9(1) 9(4)	23.3±3.3 22.5± 2.7	None	None	TIDM	None	2.75×10^6^/kg	IV	12
Yu 2011 [25]	hUC-MSCs Control	19.67±2.58 14.83±8.18	6(3) 6(2)	21.56±1.67 20.08± 3.48	10.53± 0.98 9.16± 3.30	8.23±2.12 8.08±1.83	TIDM	≦3months	1×10^7^	IV	9
Hu 2013 [26]	WJ-MSCs Control	17.6±33.69 18.2±30.60	15(6) 14(6)	20.9±14.33 21.3±15.71	6.85±2.87 6.79±3.14	5.7±1.71 5.4±1.64	TIDM	≦6months	(2.6±1.2)×10^7^	IV	24
Zhang 2016 [24]	AD-MSCs Control	22. 1 ± 6. 6 21. 6 ± 6. 8	16(7) 17(7)	20.8 ±1.9 21.1±1.9	6.76±1.25 6.71±1.43	5.92 ±1.68 6.13 ±1.49	TIDM	3.5 ±2.9 (months) 3. 7 ± 5. 5 (months)	1×10^7^/kg	IV	24
Bhansali 2017 [18]	ABM-MSCs ABM-MSCs Control	47.9±18.9 44.6±8.9 51.7±13.3	10(2) 10(3) 10(4)	28.8±4.4 27.9±4.5 26.4±3.8	6.8±0.3 6.8±0.8 6.5±0.5	5.8±0.8 6.0±0.8 6.1±1.0	T2DM	15.3±12.7(years) 10.2±1.3(years) 12.5±5.2(years)	(1.2±0.3)×10^9^	SPD artery Splenic artery	12
Guan 2015 [17]	hUC-MSCs	40.5 ± 9.21	6(0)	23.7±0.71	8.55±1.45	None	T2DM	42.7±31.89(months)	(0.88±0.05)×10^6^	Elbow vein	33.2±2.82
Liu 2014 [19]	WJ-MSCs	52.9± 10.5	22(7)	25.1 ± 2.4	8.20 ±1.69	7.53 ± 2.67	T2DM	8.7 ± 4.3(years)	1×10^6^/kg	Spleen artery	12
Kong 2014 [28]	hUC -MSCs Control	52.75± 12.69	8(3) 10(3)	25.94± 4.11 26.75± 4.32	7.30±1.49 None	9.17± 1.92 8.42±1.25	T2DM	6.87± 4.73(years) 11.4± 8.38 (years)	1.8×10^6^/kg	IV	6
Hu 2016 [29]	WJ-MSCs Control	52.43±4.88 53.21±8.22	31(14) 30(14)	26.74±5.41 27.03±6.68	7.67±1.23 7.54±1.31	8.24±1.55 7.91±1.44	T2DM	8.93±5.67(years) 8.3±6.07 (years)	1×10^6^/kg	IV	36
Jiang 2011 [30]	PD-MSCs	66	10(3)	None	9.8±2.2	None	T2DM	3–20(years)	1.35 ×10^6^/kg	IV	≧3

*Abbreviations: BMI* body mass index, *HbA1c* glycated hemoglobin, *FBG* fasting blood glucose, *DM* diabetes mellitus, *T1DM* type 1 diabetes, *T2DM* type 2 diabetes, *SPD* superior pancreaticoduodenal artery, *hBM-MSCs* human bone marrow mesenchymal stem cells, *hUC-MSCs* human umbilical cord mesenchymal stem cells, WJ-*MSCs* Wharton’s jelly mesenchymal stem cells, *AD-MSCs* adipose mesenchymal stem cells, *PD-MSCs* placenta-derived mesenchymal stem cells

### Effects of stem cell therapy on HbA1c of patients with DM

**Fig. 2:**
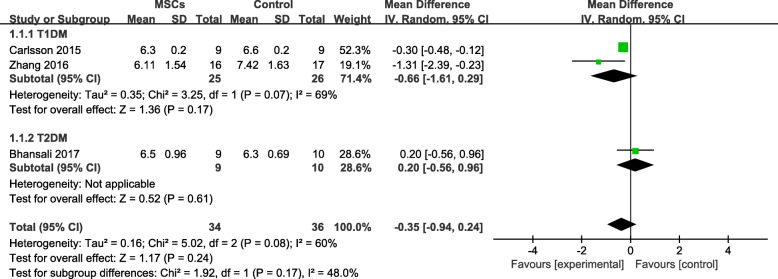
HbA1c changes in the MSC-treated and control groups after 12 months

Four trials [18, 23–25] had experimental and control groups, and one of them [25] had a follow-up period of less than 12 months. Therefore, we analyzed three [18, 23, 24] HbA1c tests. We observed that the HbA1c level was lower in the MSC-treated group with T1DM than in the control group after 12 months, but the difference was not statistically significant (MD = −0.66, 95% CI −1.61 to 0.29, *P* = 0.17; Fig. 2); however, the HbA1c level of the MSC-treated group with T2DM was slightly higher than that of the control group.

**Fig. 3:**
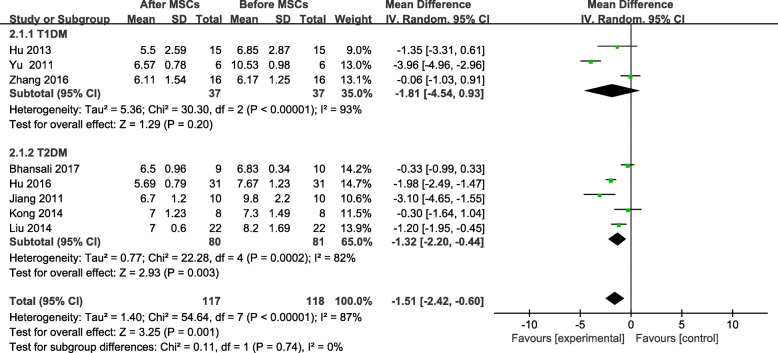
HbA1c changes after MSC therapy and individual follow-up

The HbA1c in the MSC-treated group showed a significant decrease from its baseline level, at the end of the follow-up period (MD = −1.51, 95% CI −2.42 to −0.60, *P*=0.001; Fig. 3). The subgroup analyses of patients with T1DM and T2DM showed no significant change in HbA1c in the patients with T1DM after receiving MSC therapy (MD = −1.81, 95% CI −4.54 to 0.93, *P* = 0.20; Fig. 3). A significant change in the HbA1c level of the MSC-treated patients with T2DM was found after treatment with MSC therapy (MD = −1.32, 95% CI −2.20 to −0.44, *P* = 0.003, Fig. 3); however, significant changes were not found at the 3- or 12-month follow-ups (Table 2).

**Table 2:** HbA1c levels of patients with T2DM at 3, 6, and 12 months after MSCs therapy

Follow-up (months)	Test for heterogeneity		Analysis model	Test for overall effect		WMD or SMD	95% CI
	*I*^2^ (%)	*P*		*Z*	*P*		
3	87	0.006	Random	0.89	0.37	−0.60	(−1.92, 0.72)
6	85	<0.0001	Random	2.60	0.009	−1.30	(2.27, −0.32)
12	66	0.09	Random	1.72	0.09	−0.75	(−1.60, 0.11)

*Abbreviations: HbA1c* glycated hemoglobin, *T2DM* type 2 diabetes, *MSCs* mesenchymal stem cells, *WMD* weighted mean difference, *SMD* standard mean difference

### Effects of stem cell therapy on fasting blood glucose (FBG)

**Fig. 4:**
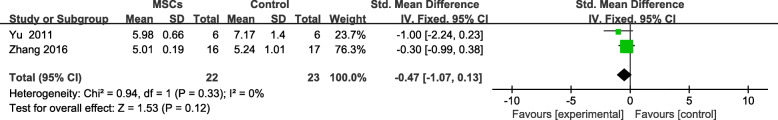
FBG changes in the MSC-treated and control groups after individual follow-up

**Fig. 5:**
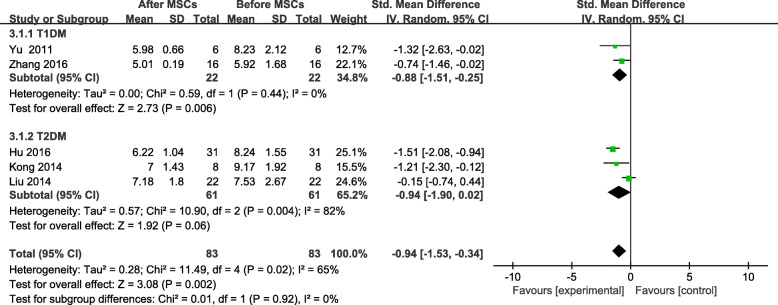
FBG changes after MSC therapy and individual follow-up

Six trials reported FBG levels and two studies [24, 25] had control groups and data that could be extracted. At the end of the follow-up period, no significant difference was found between the MSC-treated group and the control group (SMD = −0.47; 95% CI −1.07 to 0.13, *P* = 0.12; Fig. 4). The FBG level of MSC-treated patients decreased significantly from baseline to the end of the follow-up period (SMD = −0.94, 95% CI −1.53 to −0.34, *P* = 0.02; Fig. 5). The FBG of MSC-treated patients with T1DM showed a significant change by the end of the follow-up period (SMD = -0.88, 95% CI −1.51 to −0.25, *P* = 0.006; Fig. 5), but no significant difference was found in patients with T2DM (SMD = −0.94, 95% CI −1.90 to 0.02, *P* = 0.06; Fig. 5). Similarly, no significant changes in FBG levels were found at the 6- or 12-month follow-ups (Table 3).

**Table 3:** FBG levels of patients with T2DM at 6 and 12 months after MSC therapy

Follow-up (months)	Test for heterogeneity		Analysis model	Test for overall effect		WMD or SMD	95% CI
	*I*^2^ (%)	*P*		*Z*	*P*		
6	58	0.09	Random	0.73	0.46	−2.8	(−1.02, 0.47)
12	84	0.01	Random	0.72	0.47	0.54	(−0.93, 2.00)

*Abbreviations: FBG* fasting blood glucose, *T2DM* type 2 diabetes, *MSCs* mesenchymal stem cells, *WMD* weighted mean difference, *SMD* standard mean difference

### Effects of stem cell therapy on postprandial blood glucose (PBG)

**Fig. 6:**
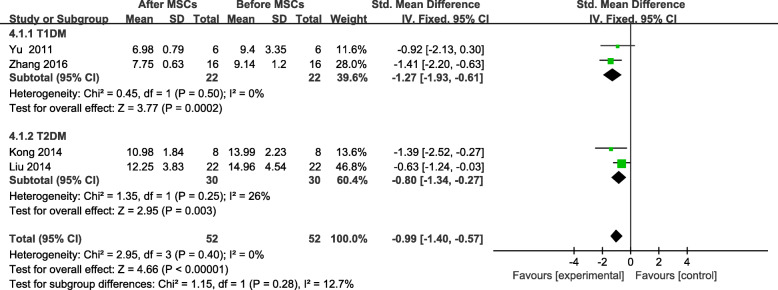
PBG changes after MSC therapy and individual follow-up

The PBG level was analyzed in four of the included trials involving 52 patients [19, 24, 25, 28]. The PBG of the MSC-treated group was significantly lower at the end of the follow-up period (SMD = −0.99, 95% CI −1.40 to −0.57, *P* < 0.00001; Fig. 6). The FBG reduction in the participants with T2DM (SMD = −0.80, 95% CI −1.34 to −0.27, *P* = 0.01; Fig 6) was significantly smaller than that of the patients with T1DM, and the PBG of the MSC-treated group with T1DM decreased significantly by the end of the follow-up period (SMD = −1.27, 95% CI −1.93 to −0.61, *P* = 0.0002; Fig. 6).

### Effects of stem cell therapy on fasting C-peptide (F-CP)

**Fig. 7:**
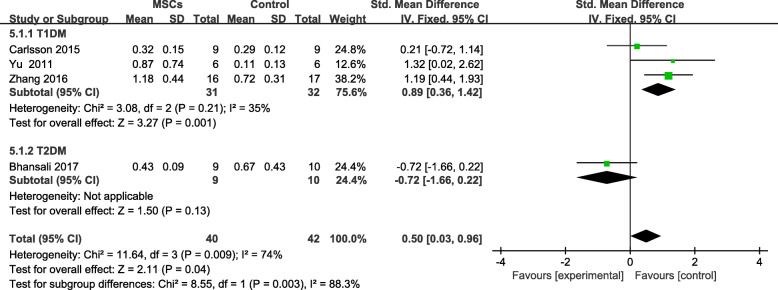
F-CP changes in the MSC-treated and control groups after individual follow-up

**Fig. 8:**
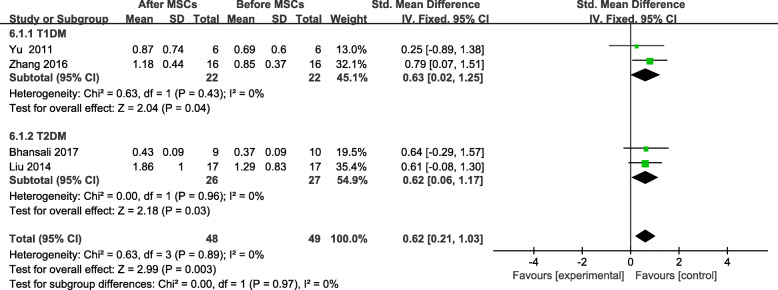
F-CP changes after MSC therapy and individual follow-up

Four trials [18, 23–25] reported F-CP levels; however, no significant difference was found between the MSC-treated group and the control group at the end of the follow-up period (SMD = 0.50; 95% CI 0.03 to 0.96, *P* = 0.04; Fig. 7). The F-CP level of the participants with T1DM was higher in the MSC-treated group than in the control group (SMD = 0.89, 95% CI 0.36 to 1.42, *P* = 0.001; Fig. 7). The F-CP level was also lower in the MSC-treated patients with T2DM than in the controls, and its level increased significantly from baseline, in the MSC-treated group after follow-up (SMD = 0.62, 95% CI 0.21 to 1.03, *P* = 0.003; Fig. 8). The MSC-treated group was found to have significant increases in the F-CP level after 6 and 12 months of follow-up (Table 4).

**Table 4:** F-CP levels of patients with T2DM at 3, 6, and 12 months after MSCs therapy

Follow-up (months)	Test for heterogeneity		Analysis model	Test for overall effect		WMD or SMD	95% CI
	*I*^2^ (%)	*P*		*Z*	*P*		
3	29	0.24	Fixed	1.65	0.10	0.46	(−0.09, 1.01)
6	42	0.19	Fixed	2.94	0.003	0.85	(0.29, 1.42)
12	0	0.96	Fixed	2.18	0.03	0.62	(0.06, 1.17)

*Abbreviations: F-CP* fasting C-peptide, *T2DM* type 2 diabetes, *MSCs* mesenchymal stem cells, *WMD* weighted mean difference, *SMD* standard mean difference

### Effects of stem cell therapy on insulin requirements of patients with DM

**Fig. 9:**
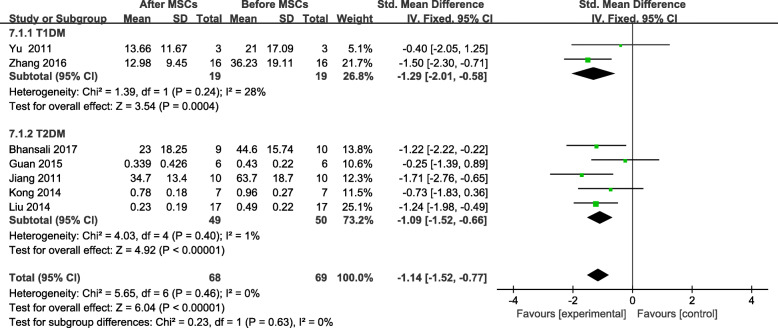
Changes in the insulin requirements of patients with DM after MSC therapy and individual follow-up

Seven trials included information on the insulin requirements of 74 patients ^[18, 19 , 24, 25, 27, 28, 30]^ at the end of the follow-up period. A significant change from the baseline insulin requirement of the MSC-treated group was found (SMD = −1.14, 95% CI −1.52 to −0.77, *P* < 0.00001; Fig. 9). A significant decrease was found in the insulin requirements of the MSC-treated group with T2DM after 3, 6, and 12 months of follow-up (Table 5).

**Table 5:** Insulin requirements of patients with T2DM at 3, 6, and 12 months after MSC therapy

Follow-up (months)	Test for heterogeneity		Analysis model	Test for overall effect		WMD or SMD	95% CI
	*I*^2^ (%)	*P*		*Z*	*P*		
3	0	0.94	Fixed	3.4	0.0007	−0.89	(−1.40, −0.38)
6	0	0.56	Fixed	5.46	<0.00001	−1.22	(−1.66, −0.78)
12	0	0.53	Fixed	3.96	<0.0001	−1.07	(−1.60, −0.54)

*Abbreviations: T2DM* type 2 diabetes, *MSCs* mesenchymal stem cells, *WMD* weighted mean difference, *SMD* standard mean difference

In 20% (3/15) patients of type 1 diabetes insulin was discontinued, and 66.7% (8/12) patients, the daily insulin demand was reduced by more than 50% of the baseline [26]. In another type 1 diabetes study, insulin demand declined significantly from baseline [24]. In the study of type 2 diabetes, 5 studies detailed records of insulin decline [18, 19, 27, 29, 30]. In general, 37% (20/54) achieved insulin independence [19, 27, 29], and 48.5% (33/68) saw a decrease in insulin demand of more than 50% [18, 19, 29, 30].

### Adverse events

Minor hypoglycemic episodes were reported in two studies [18, 29], but none of them were severe. Nausea and vomiting were reported in 2 patients [18], mild and transient fever were observed in 6 patients during infusion in 2 studies [19, 20], and hemorrhage at the arterial puncture site was observed in 2 patients [18, 19]. No serious or chronic side effects or legacy effects were observed during follow-up, which suggests that MSCs are relatively safe in the treatment of DM.

## Discussion

Our meta-analysis of ten studies with 239 patients showed that, compared with baseline levels, significant changes were found in the HbA1c, FBG, PBG, F-CP, and insulin requirements of patients with DM after they received MSC therapy.

Some of the included studies that explored the usefulness of treatment with MSCs in patients with T2DM found a significant decrease in insulin requirements and an increase in C-peptides [13, 31]. Other studies have reported that autologous MSCs are effective in animals and patients with T1DM [32, 33]. These results are consistent with our findings, and they support MSC transplantation as an effective treatment for DM. In clinical practice, the FBG and the 2-h postprandial blood glucose are the criteria for the diagnosis of diabetes, as they reflect the function of the islet cells, although there are other influencing factors. The HbA1c level, which reflects blood glucose control for the past 8–12 weeks, is a measure of diabetes control. Our meta-analysis found that the baseline levels of FBG, PBG, and HbA1c dropped significantly after MSC therapy. The subgroup analysis found that the decrease in the FBG and PBG of participants with T1DM was more pronounced than the changes in those with T2DM; the level of HbA1c in the MSC-treated group with T1DM was lower than that of the participants in the control group after 12 months of follow-up. Hence, it was clear that MSCs had a therapeutic effect on blood glucose regulation in patients with DM, and the benefits for patients with T1DM were more pronounced.

Our meta-analysis showed an increase in the F-CP level in the MSC-treated group with T1DM, which was higher than that of the control group, and an increase in the F-CP level of the MSC-treated group with T2DM after 12 months. The F-CP level is an indicator of the insulin secretion function of pancreatic islet cells, and an increased level indicates increased insulin secretion. The increase in insulin secretion may be due to the expansion of insulin-secreting B cells, or the result of increased insulin secretion of the remaining B cells. Our results also found that 7 included studies reported a significant decrease in insulin requirements after MSC therapy, compared to baseline. Similar results were found in participants with T2DM at 3, 6, and 12 months of follow-up; however, due to limitations of the trial and lack of data, the changes in the insulin requirements of participants with T1DM at each of the follow-ups could not be calculated. In conclusion, the efficacy of MSCs in reducing insulin requirements was consistent among the included studies, and it was sustained at the end of most studies’ follow-ups. However, studies with complete data and longer follow-ups are needed. We found that MSCs are more beneficial in the treatment of T1DM than T2DM.

No significant difference was found in the HbA1c or FBG levels at the end of the follow-up between the MCS-treated group and the control group. Nor was a significant change found in the FBG level of the MSC-treated group with T2DM. The F-CP level was lower in the MSC-treated group with T2DM than it was in the control group. A possible explanation for these findings is that the sample size was small, which might have led to insufficient statistical power. In addition, the inclusion of non-randomized studies in the meta-analysis could have led to bias.

Despite encouraging results in pre-clinical studies, key issues that need to be considered before MSC-based therapies become a safe and effective option for clinical researches. The clinical efficacy of MSCs is related to cell source, treatment cycle, culture expansion protocol, passage number, timing and route of administration, dosage, donor characteristics, freshly prepared, or cryopreserved cells. At present, the clinical application cycle of MSCs is difficult to be unified, and there is no unified treatment principle in the world. There are great differences between the diagnosis and treatment for different hospitals and laboratories. They were based on a small number of trials and need confirmation in larger randomized trials.

## Conclusion

MSCs can improve the blood glucose control of patients with DM and can be used to treat DM safely and effectively in the short term, especially T1DM. However, the detection of long-term effects requires longer follow-up periods, larger sample sizes, and more trials. Furthermore, no serious adverse events or significant hypoglycemic episodes were observed in MSC-treated patients with DM in all 10 studies. Therefore, MSC transplantation is considered to be a safe treatment for DM.

## Data Availability

Not applicable

## Abbreviations

MSC: Mesenchymal stem cell
DM: Diabetes mellitus
T1DM: Type 1 diabetes mellitus
T2DM: Type 2 diabetes mellitus
CNKI: Chinese National Knowledge Infrastructure
SD: Standard deviation
SMD: Standard mean difference
WMD: Weighted mean difference
CI: Confidence interval
HbA1c: Glycated hemoglobin
F-CP: Fasting C-peptide
FBG: Fasting blood glucose
PBG: Postprandial blood glucose
